# Comparison of Conventional Polyethylene Wear and Signs of Cup Failure in Two Similar Total Hip Designs

**DOI:** 10.1155/2013/710621

**Published:** 2013-04-11

**Authors:** Thomas B. Pace, Kevin C. Keith, Estefania Alvarez, Rebecca G. Snider, Stephanie L. Tanner, John D. DesJardins

**Affiliations:** ^1^Department of Orthopaedics, Greenville Health System, Greenville, SC 29605, USA; ^2^University of South Carolina SOM, Greenville Health System, P.O. Box 27114, Greenville, SC 29616, USA; ^3^Department of Bioengineering, Clemson University, Clemson, SC 29634, USA

## Abstract

Multiple factors have been identified as contributing to polyethylene wear and debris generation of the acetabular lining. Polyethylene wear is the primary limiting factor in the functional behavior and consequent longevity of a total hip arthroplasty (THA). This retrospective study reviewed the clinical and radiographic data of 77 consecutive THAs comparing in vivo polyethylene wear of two similar acetabular cup liners. Minimum follow-up was 7 years (range 7–15). The incidence of measurable wear in a group of machined liners sterilized with ethylene oxide and composed of GUR 1050 stock resin was significantly higher (61%) than the compression-molded, GUR 1020, O_2_-free gamma irradiation sterilized group (24%) (*P* = 0.0004). Clinically, at a 9-year average followup, both groups had comparable HHS scores and incidence of thigh or groin pain, though the machined group had an increased incidence of osteolysis and annual linear wear rate.

## 1. Introduction

Ultra high molecular weight polyethylene (UHMWPE) is currently the most widely used polymer for joint replacement prosthesis. Polyethylene mm head were used in all cases. The ceramicwear is the primary limiting factor in the functional behavior and consequent longevity of a total hip arthroplasty (THA). Polyethylene debris has been often linked to the development of osteolysis with subsequent loss of bone stock and implant fixation [1]. Linear wear rate of polyethylene is closely associated with osteolysis following THA, more so than patient weight, femoral head material, or implant design and offset. Acetabular cup loosening due to polyethylene wear is the most frequent reason for long-term revision in THAs, especially in young and active patients [2, 3].

Multiple factors have been identified as contributing to polyethylene wear and debris generation of acetabular lining. These variables include conformity of the articulating surface, polyethylene thickness, femoral head diameter, polyethylene locking mechanism, polyethylene additives [4], sterilization technique [5, 6], manufacturing method [4, 7], and surgical implantation technique [8]. Linear penetration of the femoral head into the polyethylene occurs through creep and wear. Creep occurs at a rate of approximately 0.18–0.2 mm/year and subsides after approximately the first 24 months. The remaining penetration is considered wear from multifactorial sources and typically occurs at reported rates of 0.05–0.18 mm/year for conventional polyethylene in THA, with compression molding having comparatively less wear [9–11]. This study compares linear wear rate of two similar total hip systems that are able to reduce the variables of interest to polyethylene resin, sterilization technique, and manufacturing methods.

The acetabular polyethylene liners of one of the total hip systems were machine fabricated using a bar extrusion technique from GUR 1050 stock resin, before being sterilized with ethylene oxide (EtO). This group will henceforth be referred to as Group 1. The other hip design uses compression molded polyethylene, from GUR 1020, resin and was sterilized using O_2_-free gamma irradiation. This grouping of polyethylene liners will henceforth be referred to as Group 2. The two hip systems had similar polyethylene locking mechanisms, articulating head surfaces, and articulating head geometry. 

In the past decade, there has been significant progress in the development of a more wear and oxidation resistant UHMWPE alternative such as the highly cross-linked UHMWPE. Initial lab and clinical studies have shown that cross-linked polyethylene may be more wear resistant than non cross-linked alternatives [12]. The focus of this study, however, compares two conventional (non highly cross-linked GUR 1050 and 1020) polyethylene liners commonly used at the time the arthroplasties were performed. The goal of this study was to clinically and radiographically compare these two similar acetabular cup liners which differ primarily in machine versus molded with respect to their linear polyethylene wear. There is no associated conflict of interests for any author listed for this study.

## 2. Materials and Methods

Institutional Review Board approval was granted for this study. Criteria for this study included THA cases with at least 7-year followup, with standard polyethylene (with a minimum of 7 mm thickness), with the same diameter femoral heads 28 mm Alumina Ceramic (Al203) or Cobalt Chrome (CoCr), and one of two press fit titanium acetabular cups. The CoCr femoral heads of both groups were from the same manufacturer. Seventy-seven THAs, performed by the same surgeon that occurred between February 1996 and August 2003, with these criteria were retrospectively reviewed. Clinical and radiographic data-collected including presurgery diagnosis, patient demographics, pre- and postsurgery Harris Hip Scores (HHS), incidence and severity of polywear, osteolysis, and patient reporting thigh and/or groin pain. Refer to Table 1 for demographical information.

This study compares clinical and radiographic assessment of polyethylene wear of two different acetabular cup liners, Group 1 (Smith and Nephew, Memphis, TN, USA) and Group 2 (Zimmer, formerly Sulzermedica, Warsaw, IN, USA). Each liner studied articulates with either a cobalt chrome femoral head (Zimmer, formerly Sulzermedica, Warsaw, IN, USA) or a ceramic femoral head (Alumina) (CeramTec, Germany) and has effective and similar locking mechanisms within the acetabular cup to minimize backside wear.

All surgeries were performed using a posterolateral approach. Thirty-one press fit Smith and Nephew Reflection acetabular cups (Group 1) and 46 press fit Zimmer Intraop acetabular cups (Group 2) were implanted. Cup screws were used based on surgical indication for cup fixation and stability. The natural hip press fit stem and a corresponding CoCr or Al_2_O_3_ 28 mm head were used in all cases. The ceramic heads were used primarily in younger male patients with physically demanding jobs. Postoperative rehabilitation included patients being weight-bearing as tolerated with walker support for 3–6 weeks as needed. Venous thrombosis event (VTE) prophylaxis was based on an individual patient VTE risk assessment. For the standard at risk patient included oral warfarin 5 mg the night of surgery and continued daily until the ProTime (prothrombin time) reached 15 seconds or the INR (international normalized ratio) reached 1.2–2. Oral warfarin 2 mg was then given daily for a 4-week protocol of 2 mg per day mini-fixed dose oral warfarin regimen. For the patient without higher VTE or bleeding risk assessment, once the hospital ProTime reached 15 seconds or INR (International Normalized Ratio) levels reached 1.2–2.0, post discharge monitoring was not done unless signs or symptoms of bleeding occurred. For higher risk VTE patients, higher dose monitored oral warfarin was used (ProTime of 18–20 seconds or INR range 2.0–2.5). All patients were counseled to avoid dislocation-prone lower extremity positioning of surgical leg internal rotation and adduction and maintain less than 90 hip flexion for 12 weeks following surgery.

Radiographic assessment of osteolysis was performed in each case at the most recent annual clinical follow up-period and classified based on Gruen et al.'s and DeLee and Charnley's classification for the femoral stem and acetabulum respectively [13, 14].

Linear polywear rate and cup abduction/inclination angle were measured on digital radiographs using femoral head size to standardize magnification. Linear wear was assessed with a resolution of 0.1 mm using techniques described and validated by Griffith et al. and Livermore et al. [15, 16]. Linear polyethylene wear was calculated from the most recent AP pelvis X-ray by subtracting the shortest distance from the femoral head to the (superior) inner cup from the original polyethylene thickness. In each case, this calculated difference was adjusted for X-ray magnification using the 28 mm femoral head as a reference. The magnification range varied from 14% 26%. Limitations of this technique do not allow for assessment of volumetric wear. Patients with ≤1 mm of head penetration on AP X-rays were categorized as having progressing creep only and not listed as measurable polyethylene wear. As all patients had a minimum of 7-year follow-up, the polyethylene deformation secondary to creep was felt to be non-contributory after the first two-year wear in period and equal in both study groups. The data was analyzed using Fishers exact probability test (*α* = 0.05).

**Figure 1:**
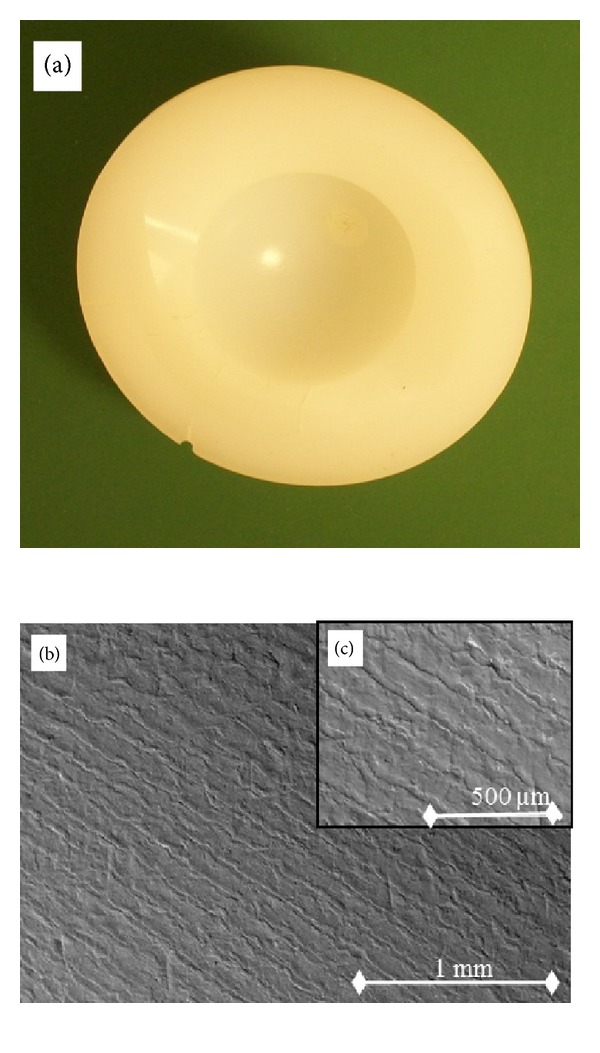
(a) The machined polyethylene acetabular cup retrieved at 5.5 years in this study paired with a cobalt chrome head. A photomicrograph of the nonarticulating surface of the retrieved conventional UHMWPE machined liner (b) 40x and (c) 95x (backscattered Topographical mode).

**Figure 2:**
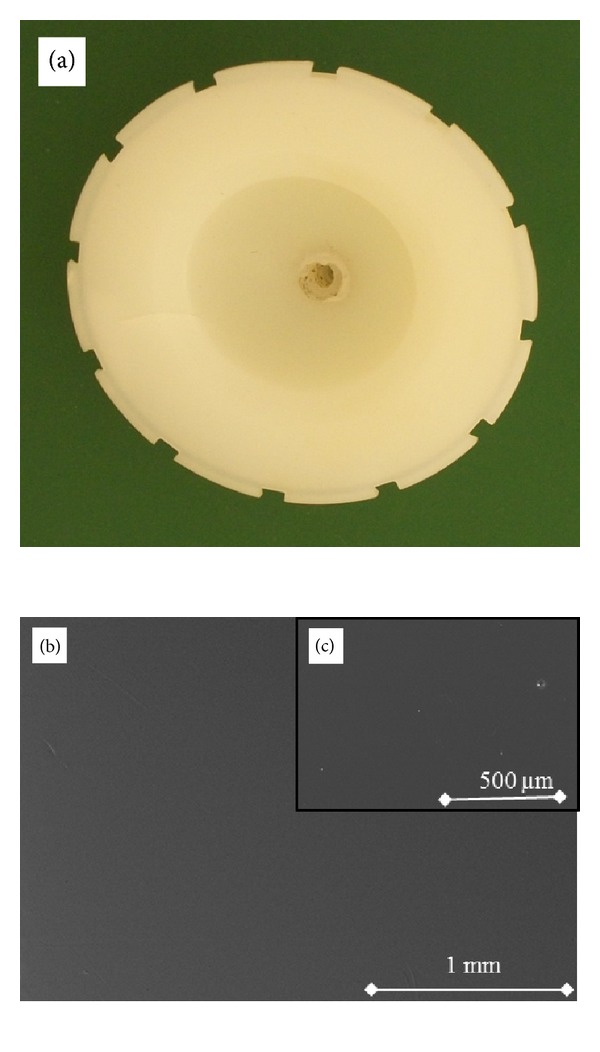
(a) The retrieved compression-molded polyethylene acetabular cup retrieved at 7.2 years (in) this study paired with a cobalt chrome femoral head. A photomicrograph of the non-articulating surface of the retrieved UHMWPE compression molded (b) 40x and (c) 95x (backscattered topographical mode).

Both titanium cup designs incorporated secure polyethylene inserts with locking mechanisms that significantly restrict both rotational and pistoning motions to minimize backside wear potential. Both cups incorporate hemispherical geometry with roughened outer surfaces for acetabular bone ingrowth. All polyethylene liners were a minimum of 7 mm actual thickness with ranges from 7 to 15.5 mm depending on the size of the individual patient's acetabulum and subsequent cup diameter. The acetabular cups liners differ primarily with respect to stock resin, manufacturing methods, and sterilization techniques (Table 2).  Figure 1 shows a photograph of an explanted machined polyethylene and corresponding scanning electron microscope (SEM) image.  Figure 2 shows a photograph of an explanted compression molded polyethylene and corresponding SEM.

SEM images were taken using a variable pressure Hitachi 3400 SEM at 20 keV accelerating potential capability at magnification of 40x and 95x. It is equipped with Phillips Secondary Electron Detector 6765/50 complimented by a Hitachis Backscattered Electron.

The effect of the manufacturing procedure on the surface of the UHMWPE liners (machined and compression molded) was evaluated using noncontact profilometry using a WYKO NT2000 profilometer (Veeco Corp., Tucson, AZ, USA). Surface characterization was performed after retrieval at a nominal magnification of 25x (field of view 736 × 480 nm, ±0.1 nm). To best capture the manufactured original surface, Nonarticulating surfaces were characterized for roughness measures arithmetic surface roughness (Ra), average maximum profile peak height (Rpm), and average maximum profile valley height (Rvm). Five measurements were taken on the nonarticulating surface of each polyethylene liner in a linear fashion in order to fully quantify and characterize the component and ensure reliable and repeatable estimate of nonarticulating surface roughness. Statistical analysis (Student's *t*-test with *α* = 0.05) was performed to evaluate whether there was a significant difference between machined and compression molded nonarticulating liner surface roughness. 

In order to characterize the surface morphology of the UHMWPE liners, both optical microscopy and SEM analysis were performed on the nonarticulating surface of the UHMWPE liners. The images were taken choosing positions along the Nonarticulating surface using a stereomicroscope (model K400P, Motic Inc., Xiamen, China) with lenses providing 6x to 50x magnification with controlled fluorescent ring illumination and a color digital camera (model infinity 2-1C, Lumenera Corp., Ottawa, ON, Canada).

## 3. Results

With the numbers in this study, no significant difference was found in patient demographics between the two groups. The incidence of measurable wear in the Group 1 (61.3%) was significantly higher than that of Group 2 (23.9%) (*P* = 0.0004). The linear wear rate of Group 1 was additionally significantly higher (2.7 times) than Group 2 polyethylene's with CoCr head group (*P* = 0.0014). The linear wear rate of Group 1 was also significantly higher (1.8 times) than the Al_2_O_3_ Group 2 (*P* = 0.0028). There was no difference in the wear rate between the two subgroups in Group 2 when comparing those with CoCr heads and those with Al_2_O_3_ (*P* = 0.071) (refer to Table 3).

**Figure 3:**
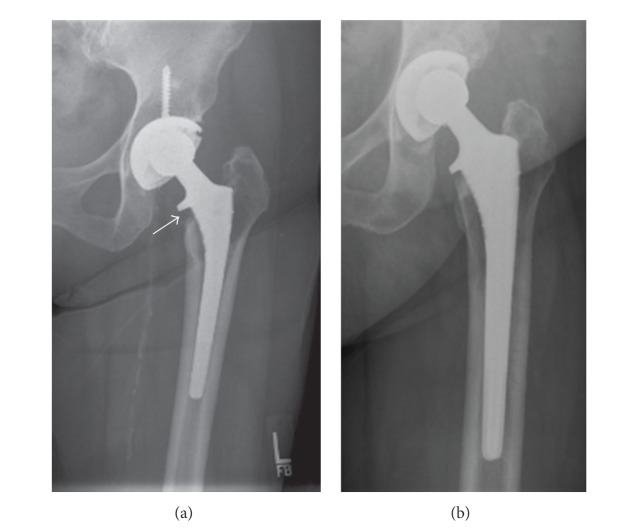
(a) AP radiograph of THA with machined polyethylene after 8 years of implantation. The measured polywear was of 4 mm. There is focal stem osteolysis and calcar erosion (arrow). (b) AP radiograph of THA with compression-molded polyethylene after 11 years of implantation. The measured polywear was of 2 mm. There were no significant signs of osteolysis.

Clinically, at a 9-year average follow-up, both groups were doing equally well with HHS scores and reported thigh or groin pain incidences of 9.7% for the machined polyethylene group and 6.5% in the compression molded group (*P* values >0.05) (refer to Table 4). The revision rate for both groups was the same, with one case in each group for recurrent dislocation. While there were no revisions for osteolysis, the incidence of osteolysis was higher in Group 1 (12.9% machined versus 0% compression molded). When assessing focal calcar erosion of ±1 cm, the incidence was higher in Group 1 (16%) than in Group 2 (2%). (refer to Table 5). Typical representative radiographs from each group are presented in Figure 3. Tables 5 and 6 present the radiographic differences in the machined polyethylene and compression molded polyethylene groups.

Nakahara et al. have shown that acetabular cups implanted with >45° abduction have higher polyethylene wear rates than cups implanted with 45° or less [8]. When assessing the cup abduction/inclination (≥45°) as a variable affecting linear wear in each group, there was not a significant difference (*P* = 0.777).

The complications in each group were comparable and no statistical difference was found in the complications in either group (refer to Table 7).

**Figure 4:**
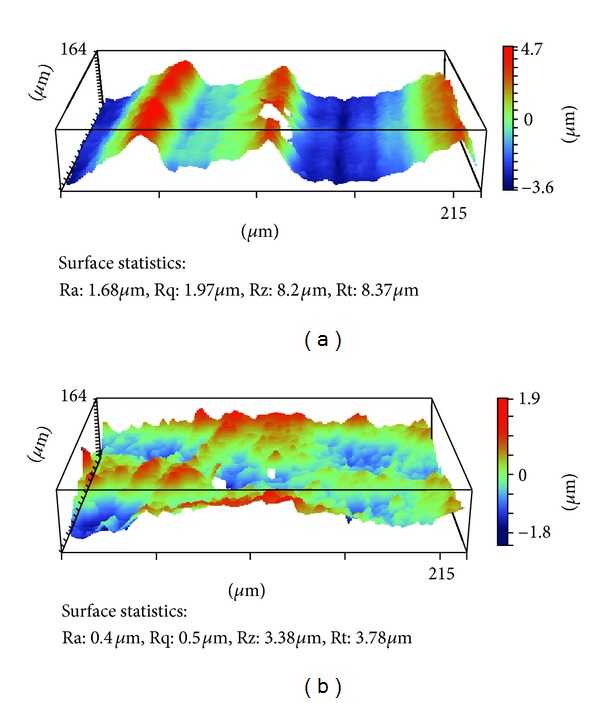
(a) Nonarticulating surface profile for machined polyethylene. The machining marks are represented by the peaks and valleys illustrated by the color scale. (25x) (b) Nonarticulating surface profile of retrieved compression-molded polyethylene, which has no significant changes in its topography.

Figure 4 shows surface profiles of the retrieved Group 1 GUR 1050 UHMWPE liner 5.5 years post implantation and Group 2 GUR 1020 UHMWPE liner 7.2 years post implantation. The 3D representative profile shows the machining and compression molded marks as a result of the manufacturing process.

## 4. Discussion

This study examines the clinical polyethylene wear characteristics of two independent total hip systems. Upon comparison, significant statistical differences are found between the polyethylene wear seen within the two systems. 

The polyethylene of Group 1, made of GUR 1050 resin, sterilized through the use of ethylene oxide, was manufactured by ram extrusion and machining. Group 2 polyethylene is compression-molded from GUR 1020 resin and is sterilized by means of gamma irradiation in an oxygen free-environment. In vitro studies on tibial inserts for knee arthroplasties have shown that compression molded and oxygen free sterilization reports less wear than machined tibial inserts for total knee arthroplasties [11, 17, 18]. Yet little knowledge is available on the performance of THAs with regard to these same variables. 

The polyethylene of Group 2 was sterilized via oxygen-free gamma irradiation. Gamma irradiation in the presence of O_2_ has shown to increase polyethylene oxidation due to free radical generation and subsequent oxidation, consequently lowering mechanical properties, leading to rapid polyethylene wear. However, gamma irradiation performed in an oxygen free environment has been shown to negate the oxidative effects of irradiation [5]. Faris et al. demonstrated that annual linear wear rate significantly increased in a population of gamma irradiated liners in air when compared with a population of similar gamma irradiated hips in an oxygen free vacuum [5].

Each liner was also formed from two different polyethylene resins, GUR 1050 for Group 1 and GUR 1020 for Group 2. The lone difference between the two resins is the average molecular weight, which is around 3.5 × 106 g/mol for GUR 1020 and 5.5 × 106 for GUR 1050. Past studies have shown that the higher molecular weight provides better abrasive wear resistance, but only slightly. Kurtz [4] argued that both the impact strength and the wear resistance of each resin increased nonlinearly with increasing intrinsic viscosity of the resin, which is a function of average molecular weight. However, Tipper et al. performed an in vitro cyclic wear test between GUR 1020 and 1050 and demonstrated that while GUR 1050 did have greater wear resistance, it was not considered statistically important [19].

Previous authors have compared the bearing material effect on CoCr versus Al_2_O_3_ with respect to polyethylene clinical wear rate in THAs [19]. Clinically, the linear wear rate for polyethylene paired with CoCr heads ranges between 0.1 and 0.3 mm/year whereas when compared with Al_2_O_3_ the range is 0.03–0.15 mm/year [20, 21]. While not statistically significant, there was a trend towards more wear in Group 2 with Al_2_O_3_ head versus CoCr heads, though the linear wear rates reported in this study fall within the expected range. This may be explained by the practice of using Al_2_O_3_ heads in younger more active patients. While there was not a difference in average age or range, there was a tendency for more males in the compression molded Al_2_O_3_ head group. There was a trend towards younger males in the Al_2_O_3_ head group compared to either group (machined or compression molded) with CoCr. This was not statistically different (Table 1).

The final variable of interest that could significantly affect polyethylene wear is the manufacturing technique. There are various types of fabrication methods that can be employed in the fabrication of polyethylene orthopedic implants. Most of the acetabular liners used today are machined from extruded bar stock or compression-molded directly from the polyethylene powder resin [4]. Machined polyethylene liners are made by first producing a polyethylene bar stock from the resin, followed by the machining process, which cuts the bar stock to a very precise size and geometry through the use of a lathe. The polyethylene bar stocks themselves are produced through a process known as ram extrusion where the polyethylene resin is simultaneously heated and pressurized within an evacuated chamber. As the solid polyethylene forms, it is extruded through an open extrusion port within the chamber [22]. Because the extrusion process is noncontinuous, inconsistencies can be found within the solid polyethylene bar stock. These zones of polyethylene inconsistencies, or what Bankston et al. referred to as “dead zones,” can produce areas of greatly altered molecular weight resulting in altered wear resistance [22]. The alternate process of compression molding is a single-step process where the polyethylene resin is molded directly into the predetermined size and geometry of the acetabular insert.

In addition to the maldistribution of solid-phase polyethylene within the ram extruded bar stock, surface characteristics and topography may have a significant role in polyethylene oxidation. In the machined polyethylene insert, machine marks from the lathe creates numerous micron size grooves and shreds on the bearing surface, which are not found on the surface of the compression molded polyethylene insert. This was confirmed in the current study as shown in Figure 4. Bankston et al. mentioned that not only could this be a source of third body wear, but also the microindentations and scratches on the surface created added surface area to which oxidation could more easily occur [22]. The characteristic surfaces seen in the three-dimensional topographic images of Figure 4 are a result of the manufacturing methods of each polyethylene design. The repeating peak and valley topography in Figure 4 is indicative of the machining marks, which occur during the machining portion of liner formation. The compression molded topographic image in comparison lacks the large surface wave ranges and is flatter owing to the forming process. The micron-level surface differences between each polyethylene cause a higher surface roughness average (Ra) in the machined polyethylene. The representative images show an Ra of 1.68 *μ*m for machined and 0.40 *μ*m for molded surfaces, which are characteristic of these surfaces in UHMWPE implants [12]. Despite the SEM and surface profile findings noted, the gross inspection of each retrieval specimen showed no visible signs of delamination, pitting, scratches, or cracking.

## 5. Conclusion

In summary, the implant design using GUR1050 bar stock, sterilized in ETO, with final articular surface geometry machined had significantly more linear wear and radiographic osteolysis. The implant using GUR 1020 stock powder, compression-molded into final surface geometry without machining, and sterilized in inert gamma irradiation showed significantly less radiographic measured wear and osteolysis. It is beyond the scope of this paper to conjecture which of the differing elements is responsible for the greater linear wear rate in one implant versus the other. PE wear rates have been shown to predict osteolysis, implant longevity, and revision surgery, and it is routine practice to monitor all implants for wear and signs of failure. Most current THA implants are using highly cross-linked PE because of more favorable reported wear rates. However, there are a large number of hip arthroplasty implants using conventional PE that were implanted before highly cross-linked PE was available. Cross-linked PE has also been slow to move into wide-scale implementation on a global scale due to its high cost and low availability outside of the United States. Thus, understanding the fundamental core materials and the manufacturing process for conventional PE implants and having comparative clinical reviews as described here suggest that it may be prudent to monitor some implants more closely than others for polyethylene wear-related signs of problems.

## Figures and Tables

**Table 1:** Patient demographical information for the different manufactured polyethylene groups with different material pairings.

	Group 1	Group 2	
	CoCr	CoCr	Al_2_0_3_
Total cases	**31**	**15**	**31**
Males	13	4	17
Females	18	11	14
Median age* (range)	66 (46–86)	65 (38–81)	57 (42–77)
Diagnosis			
Osteoarthritis	31	15	27
AVN**	0	0	2
Other***	0	0	2

*Age at time of surgery.

**AVN: avascular necrosis.

***Dysplasia and rheumatoid arthritis.

**Table 2:** Comparison of the characteristics of the acetabular polyethylene.

	Group 1	Group 2
Stock resin	GUR 1050	GUR 1020
Manufacturing process	Ram extrusion, final geometry machined	Compression molding
Sterilization	Ethylene oxide	Gamma irradiation, oxygen-free environment
Polyethylene thickness	Range: 7.0–15.5 mm	Range: 7.0–14.5 mm

**Table 3:** Incidence and polyethylene wear rates for both groups of material pairings used in this study.

	Group 1 (CoCr heads)	Group 2 (CoCr heads)	Group 2 (Al_2_O_3 _heads)
Incidence of >1 mm polywear	19/31	1/15	10/31
Average wear rate (range)	0.24 mm/year (0.11–0.88)	0.09 mm/year (0.09)	0.13 mm/year (0.08–0.25)

**Table 4:** Clinical results for hip scores and pain for both groups material pairings used in this study.

	Group 1	Group 2	
	(CoCr heads)	(CoCr heads)	(Al_2_O_3_ heads)
Total	**31**	**15**	**31**
Preoperation HHS Average (range)	67 (58–87)	68 (58–87)	67 (58–81)
Last clinical HHS Average (range)	99.1 (95–100)	98.9 (93–100)	99.5 (95–100)
Thigh pain	2	1	0
Groin pain	1	0	2

**Table 5:** Radiographic findings for each acetabular liner articulating against Same CoCr bearing counterface.

	Group 1 (CoCr heads)	Group 2 (CoCr heads)	*P* values
Total	**31**	**15**	
Calcar erosion	5	1	0.65
Osteolysis	4	0	0.29
Cup	1	0	1
Stem	3	0	0.54

**Table 6:** Radiographic results for each acetabular liner articulating against different femoral head material.

	Group 1 (CoCr heads)	Group 2 (Al_2_O_3_ heads)	*P* values
Total	**31**	**31**	
Calcar erosion	5	0	0.05
Osteolysis	4	0	0.11
Cup	1	0	1
Stem	3	0	0.24

**Table 7:** Reported clinical complications for the seventy-seven THR considered in this study.

	Group 1	Group 2	
	(CoCr heads)	(CoCr heads)	(Al_2_O_3_ heads)
Total	**31**	**15**	**31**
Total complications	2	3	3
Dislocation	1	2	2
Infection	0	1	0
Symptomatic DVT & PE	0	0	0
Major/minor bleeding	0	0	0
Heterotopic ossification	1 (grade 2)	0	1
Revisions	1 (recurrent dislocation)	1 (recurrent dislocation)	0
